# Bis[8-ethyl-5-oxo-2-(piperazin-4-ium-1-yl)-5,8-dihydro­pyrido[2,3-*d*]pyrimidine-6-carb­oxy­lic acid] 2,5-dicarb­oxy­benzene-1,4-di­carboxyl­ate octa­hydrate

**DOI:** 10.1107/S1600536811011068

**Published:** 2011-03-31

**Authors:** Guang-Ju Zhang, Jiang-Hong He, Shi-Wei Yan, Zhong-Li Ye, Guang-Hua Xin

**Affiliations:** a College of Chemistry and Chemical Engineering, Southwest University, Chongqing,400715, People’s Republic of China

## Abstract

The asymmetric unit of the title compound, 2C_14_H_18_N_5_O_3_^+^·C_10_H_5_O_8_^2−^·8H_2_O, contains one [H_2_ppa]^+^cation, one half of an [H_2_btec]^2−^ anion (H_4_btec = 1,2,4,5-benzene­tetra­carb­oxy­lic acid and Hppa = 8-ethyl-5-oxo-2-piperazin-1-yl-5,8-dihydro­pyrido[2,3-*d*]pyrimidine-6-carb­oxy­lic acid) that is completed by inversion symmetry and four water mol­ecules. In the crystal, the mol­ecules are connected by inter­molecular hydrogen-bonding inter­actions and π–π stacking between the benzene rings of the [H_2_btec]^2−^ anion and the pyrimidine rings of the [H_2_ppa]^+^ cation [centroid–centroid distance = 3.597 (3) Å], generating a three-dimensional supra­molecular structure.

## Related literature

For general background to the use of quinolones in the treatment of infections, see: Mizuki *et al.* (1996 ▶).
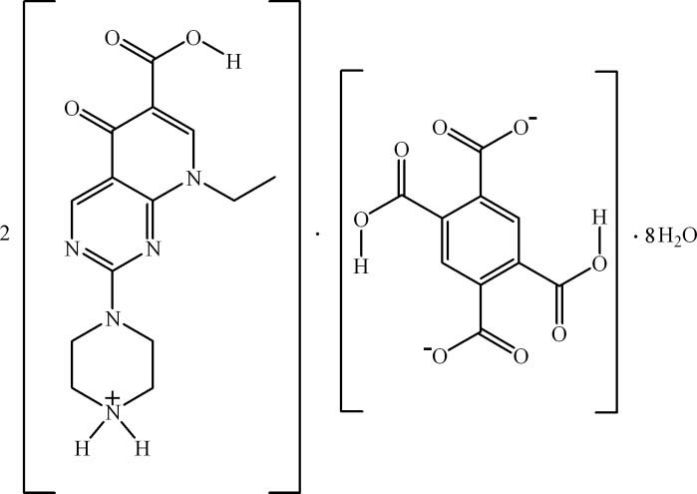


## Experimental

### 

#### Crystal data

C_14_H_18_N_5_O_3_^+^·0.5C_10_H_4_O_8_^−^·4H_2_O
*M* *_r_* = 502.46
Triclinic, 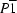

*a* = 8.8336 (16) Å
*b* = 11.103 (2) Å
*c* = 12.445 (2) Å
α = 83.010 (2)°
β = 76.737 (2)°
γ = 73.831 (2)°
*V* = 1138.9 (4) Å^3^
*Z* = 2
Mo *K*α radiation
μ = 0.12 mm^−1^
*T* = 296 K
0.52 × 0.48 × 0.39 mm

#### Data collection

Bruker SMART APEX CCD area-detector diffractometer
Absorption correction: multi-scan (*SADABS*; Sheldrick, 1996 ▶) *T*_min_ = 0.940, *T*_max_ = 0.954
10252 measured reflections
5071 independent reflections
3550 reflections with *I* > 2σ(*I*)
*R*_int_ = 0.021

#### Refinement

*R*[*F* ^2^ > 2σ(*F* ^2^)] = 0.046
*wR*(*F* ^2^) = 0.160
*S* = 1.01
5071 reflections
352 parameters
14 restraints
H atoms treated by a mixture of independent and constrained refinement
Δρ_max_ = 0.33 e Å^−3^
Δρ_min_ = −0.20 e Å^−3^

### 

Data collection: *SMART* (Bruker, 2001 ▶); cell refinement: *SAINT* (Bruker, 2001 ▶); data reduction: *SAINT*; program(s) used to solve structure: *SHELXS97* (Sheldrick, 2008 ▶); program(s) used to refine structure: *SHELXL97* (Sheldrick, 2008 ▶); molecular graphics: *SHELXTL-Plus* (Sheldrick, 2008 ▶); software used to prepare material for publication: *SHELXL97*.

## Supplementary Material

Crystal structure: contains datablocks I, global. DOI: 10.1107/S1600536811011068/ff2005sup1.cif

Structure factors: contains datablocks I. DOI: 10.1107/S1600536811011068/ff2005Isup2.hkl

Additional supplementary materials:  crystallographic information; 3D view; checkCIF report

## Figures and Tables

**Table 1:** Hydrogen-bond geometry (Å, °)

*D*—H⋯*A*	*D*—H	H⋯*A*	*D*⋯*A*	*D*—H⋯*A*
O6—H6*A*⋯O4^i^	0.92 (2)	1.47 (2)	2.392 (2)	178 (3)
N1—H1*A*⋯O*W*1^ii^	0.90	2.08	2.952 (2)	164
N1—H1*A*⋯O6^ii^	0.90	2.56	3.022 (2)	113
N1—H1*B*⋯O*W*2^ii^	0.90	1.82	2.717 (2)	176
O*W*1—H*W*1*A*⋯O1^iii^	0.88 (2)	1.98 (2)	2.780 (2)	150 (2)
O*W*1—H*W*1*B*⋯O3	0.85 (2)	2.38 (2)	3.041 (2)	135 (2)
O*W*1—H*W*1*B*⋯O7	0.85 (2)	2.57 (2)	3.204 (2)	132 (2)
O*W*1—H*W*1*B*⋯O6	0.85 (2)	2.59 (2)	3.084 (2)	118 (2)
O2—H2*A*⋯O3	0.99 (2)	1.56 (2)	2.5013 (19)	159 (2)
O*W*2—H*W*2*B*⋯O*W*4^iv^	0.86 (2)	1.86 (2)	2.703 (3)	168 (3)
O*W*2—H*W*2*A*⋯O5^iv^	0.84 (2)	1.98 (2)	2.819 (2)	173 (3)
O*W*3—H*W*3*A*⋯O*W*1^v^	0.85 (2)	1.93 (2)	2.770 (2)	169 (3)
O*W*3—H*W*3*B*⋯O2^vi^	0.85 (2)	2.17 (2)	3.011 (3)	172 (3)
O*W*4—H*W*4*A*⋯O4^vii^	0.87 (2)	1.94 (2)	2.782 (2)	163 (3)
O*W*4—H*W*4*B*⋯O*W*3	0.81 (2)	1.97 (2)	2.775 (3)	174 (3)

Symmetry codes: (i) 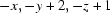; (ii) 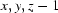; (iii) 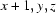; (iv) 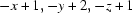; (v) 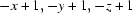; (vi) 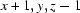; (vii) 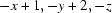.

**Fig. 1.**
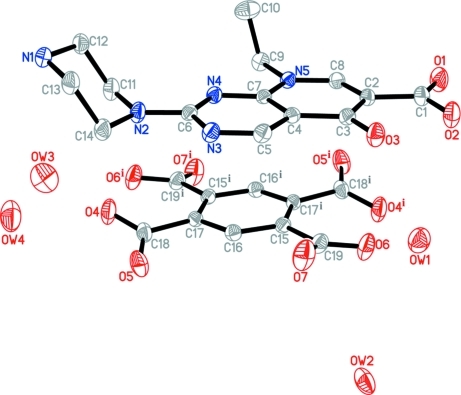
The structure of 1. Displacement ellipsoids are drawn at the 30% probability level. H atoms have been omitted for clarity. [Symmetry code: -x,-y+2, -z+1].

## supplementary crystallographic information

### Comment

Pipemidic acid (8-ethyl-5-oxo-2-piperazin-1-yl-5,8-dihydropyrido[2,3-d]pyrimidine-6- carboxylic acid) is member of quinolones used to treat infections (Mizuki *et al.*, 1996). The complexes of the Hppa and H_4_btec have not been reported till now. In this paper, the structure of the title compound, 1, is described (Fig. 1). The asymmetric unit contains one [H_2_ppa]^+^cation, one half of [H_2_btec]^2-^ anion that is completed by inversion symmetry, and four lattice water molecules. The molecules are linked by intermolecular N—H···O and O—H···O hydrogen-bonding interactions (N···O = 2.717 (2)–3.022 (2) Å, O···O = 2.392 (2)–3.204 (2) Å) and π—π stacking between the benzene rings of [H_2_btec]^2-^ anion and the pyrimidine rings of [H_2_ppa]^+^cation (centroid distance of 3.597 (3) Å) to form a three-dimensional supramolecular structure.

### Experimental

A mixture of AgNO_3_ (0.085 g, 0.5 mmol), Hppa (0.089 g, 0.25 mmol), H_4_btec (0.064 g, 0.25 mmol) and distilled water (8 ml) was stirred for 20 min. in air. The mixture was then transferred to a 18 ml Teflon-lined hydrothermal bomb. The bomb was kept at 393 K for 96 h under autogenous pressure. Upon cooling, colorless blocks of 1 were obtained from the reaction mixture.

### Refinement

The H atoms bonded to C atoms were positioned geometrically and refined using a riding model approximation [aromatic C—H = 0.93 Å, aliphatic C—H =0.96 —0.97 Å], with *U*_iso_(H) = 1.2—1.5 *U*eq(C).The H on N atoms were located in difference Fourier maps, and refined with distances restraint of N—H = 0.90 Å, and with *U*_iso_(H)= 1.2 *U*_eq_(N). The H atoms bonded to O atoms were located in difference Fourier maps and refined with *U*iso(H) = 1.3 *U*eq(O) for carboxyl groups of [C_14_H_18_O_3_N_5_]^+^and [C_10_H_4_O_8_]^2^^-^respectively. The O—H bonds are 0.986 Å and 0.924 Å in carboxyl groups of [C_14_H_18_O_3_N_5_]^+^ and [C_10_H_4_O_8_]^2^^-^. The H atoms bonded to OW atoms were located in a difference Fourier maps and refined with OW—H =0.812 Å—0.878 Å and *U*_iso_(H) = 1.1—1.5 *U*_eq_(OW).

### Crystal data

**Table:**

C_14_H_18_N_5_O_3_^+^·0.5C_10_H_4_O_8_^−^·4H_2_O	*Z* = 2
*M**_r_* = 502.46	*F*(000) = 530
Triclinic, *P*1	*D*_x_ = 1.465 Mg m^−^^3^
Hall symbol: -P 1	Mo *K*α radiation, λ = 0.71073 Å
*a* = 8.8336 (16) Å	Cell parameters from 10252 reflections
*b* = 11.103 (2) Å	θ = 2.5–27.4°
*c* = 12.445 (2) Å	µ = 0.12 mm^−^^1^
α = 83.010 (2)°	*T* = 296 K
β = 76.737 (2)°	Block, colourless
γ = 73.831 (2)°	0.52 × 0.48 × 0.39 mm
*V* = 1138.9 (4) Å^3^	

### Data collection

**Table:**

Bruker APEX CCD area-detector diffractometer	5071 independent reflections
Radiation source: fine-focus sealed tube	3550 reflections with *I* > 2σ(*I*)
graphite	*R*_int_ = 0.021
phi and ω scans	θ_max_ = 27.4°, θ_min_ = 2.5°
Absorption correction: multi-scan (*SADABS*; Sheldrick, 1996)	*h* = −11→11
*T*_min_ = 0.940, *T*_max_ = 0.954	*k* = −14→14
10252 measured reflections	*l* = −16→16

### Refinement

**Table:**

Refinement on *F*^2^	Primary atom site location: structure-invariant direct methods
Least-squares matrix: full	Secondary atom site location: difference Fourier map
*R*[*F*^2^ > 2σ(*F*^2^)] = 0.046	Hydrogen site location: inferred from neighbouring sites
*wR*(*F*^2^) = 0.160	H atoms treated by a mixture of independent and constrained refinement
*S* = 1.01	*w* = 1/[σ^2^(*F*_o_^2^) + (0.1*P*)^2^] where *P* = (*F*_o_^2^ + 2*F*_c_^2^)/3
5071 reflections	(Δ/σ)_max_ < 0.001
352 parameters	Δρ_max_ = 0.33 e Å^−^^3^
14 restraints	Δρ_min_ = −0.20 e Å^−^^3^

### Special details

**Table:**

Geometry. All e.s.d.'s (except the e.s.d. in the dihedral angle between two l.s. planes) are estimated using the full covariance matrix. The cell e.s.d.'s are taken into account individually in the estimation of e.s.d.'s in distances, angles and torsion angles; correlations between e.s.d.'s in cell parameters are only used when they are defined by crystal symmetry. An approximate (isotropic) treatment of cell e.s.d.'s is used for estimating e.s.d.'s involving l.s. planes.
Refinement. Refinement of *F*^2^ against ALL reflections. The weighted *R*-factor *wR* and goodness of fit *S* are based on *F*^2^, conventional *R*-factors *R* are based on *F*, with *F* set to zero for negative *F*^2^. The threshold expression of *F*^2^ > σ(*F*^2^) is used only for calculating *R*-factors(gt) *etc*. and is not relevant to the choice of reflections for refinement. *R*-factors based on *F*^2^ are statistically about twice as large as those based on *F*, and *R*- factors based on ALL data will be even larger.

### Fractional atomic coordinates and isotropic or equivalent isotropic displacement parameters (Å^2^)

**Table:**

	*x*	*y*	*z*	*U*_iso_*/*U*_eq_	
C1	−0.2298 (2)	0.58949 (18)	0.76095 (14)	0.0453 (4)	
C2	−0.1629 (2)	0.63420 (16)	0.64826 (13)	0.0362 (4)	
C3	0.0071 (2)	0.60173 (15)	0.60569 (13)	0.0348 (4)	
C4	0.05712 (18)	0.65003 (14)	0.49440 (12)	0.0316 (3)	
C5	0.2186 (2)	0.62709 (16)	0.43913 (13)	0.0377 (4)	
H5A	0.2968	0.5782	0.4765	0.045*	
C6	0.14597 (19)	0.74223 (16)	0.28730 (13)	0.0336 (4)	
C7	−0.05440 (18)	0.72426 (14)	0.43356 (12)	0.0295 (3)	
C8	−0.2657 (2)	0.70738 (16)	0.58476 (13)	0.0360 (4)	
H8A	−0.3755	0.7269	0.6149	0.043*	
C9	−0.33740 (19)	0.83037 (17)	0.41760 (14)	0.0402 (4)	
H9A	−0.2943	0.8957	0.3727	0.048*	
H9B	−0.4340	0.8707	0.4687	0.048*	
C10	−0.3799 (3)	0.7527 (2)	0.3443 (2)	0.0640 (6)	
H10A	−0.4572	0.8056	0.3042	0.096*	
H10B	−0.4251	0.6892	0.3887	0.096*	
H10C	−0.2847	0.7134	0.2931	0.096*	
C11	0.0817 (2)	0.85693 (19)	0.11400 (14)	0.0470 (4)	
H11A	0.1022	0.9380	0.0881	0.056*	
H11B	−0.0279	0.8712	0.1564	0.056*	
C12	0.1010 (2)	0.78182 (19)	0.01646 (14)	0.0472 (5)	
H12A	0.0685	0.7049	0.0421	0.057*	
H12B	0.0317	0.8298	−0.0325	0.057*	
C13	0.3844 (2)	0.68542 (19)	0.02854 (14)	0.0475 (5)	
H13A	0.4945	0.6720	−0.0128	0.057*	
H13B	0.3654	0.6040	0.0552	0.057*	
C14	0.3613 (2)	0.7624 (2)	0.12515 (15)	0.0474 (5)	
H14A	0.4303	0.7166	0.1751	0.057*	
H14B	0.3910	0.8403	0.0992	0.057*	
C15	0.0728 (2)	0.91872 (15)	0.58174 (12)	0.0335 (4)	
C16	0.1614 (2)	0.95494 (15)	0.48134 (14)	0.0350 (4)	
H16	0.271 (2)	0.9248 (16)	0.4737 (15)	0.037 (5)*	
C17	0.09561 (19)	1.03470 (15)	0.39901 (12)	0.0327 (4)	
C18	0.2204 (2)	1.06072 (16)	0.29829 (14)	0.0419 (4)	
C19	0.1758 (2)	0.82880 (18)	0.65566 (15)	0.0459 (4)	
N1	0.27157 (18)	0.75043 (15)	−0.04528 (11)	0.0452 (4)	
H1A	0.2821	0.7007	−0.0999	0.054*	
H1B	0.2974	0.8215	−0.0764	0.054*	
N2	0.19315 (17)	0.79002 (15)	0.18387 (11)	0.0430 (4)	
N3	0.26607 (16)	0.67052 (14)	0.33790 (11)	0.0396 (3)	
N4	−0.01218 (15)	0.77067 (13)	0.33069 (10)	0.0338 (3)	
N5	−0.21691 (15)	0.75273 (13)	0.48113 (11)	0.0333 (3)	
O1	−0.37365 (18)	0.61061 (16)	0.80048 (11)	0.0640 (4)	
OW1	0.34139 (19)	0.54945 (15)	0.80171 (12)	0.0614 (4)	
HW1A	0.4401 (19)	0.557 (2)	0.7779 (18)	0.072*	
HW1B	0.298 (2)	0.581 (2)	0.7463 (16)	0.070*	
O2	−0.12330 (19)	0.52214 (15)	0.81832 (12)	0.0622 (4)	
H2A	−0.018 (2)	0.516 (2)	0.7683 (19)	0.083 (8)*	
OW2	0.3559 (2)	0.95983 (17)	0.85270 (14)	0.0721 (5)	
HW2A	0.442 (3)	0.954 (3)	0.8054 (18)	0.097*	
HW2B	0.353 (3)	1.013 (2)	0.8988 (19)	0.103*	
O3	0.10748 (15)	0.53510 (12)	0.66146 (10)	0.0502 (3)	
OW3	0.8262 (3)	0.59914 (18)	0.05037 (17)	0.0835 (5)	
HW3A	0.764 (3)	0.562 (3)	0.0954 (17)	0.092*	
HW3B	0.840 (4)	0.570 (3)	−0.0121 (15)	0.118*	
OW4	0.6900 (2)	0.85376 (17)	0.01290 (14)	0.0782 (5)	
HW4A	0.713 (4)	0.876 (2)	−0.0572 (15)	0.107*	
HW4B	0.725 (3)	0.7783 (16)	0.022 (2)	0.092*	
O4	0.17347 (18)	1.10598 (16)	0.20881 (11)	0.0655 (4)	
O5	0.36177 (16)	1.03613 (14)	0.30721 (12)	0.0604 (4)	
O6	0.1120 (2)	0.80744 (16)	0.75738 (11)	0.0660 (4)	
H6A	0.002 (2)	0.842 (2)	0.772 (2)	0.084 (8)*	
O7	0.31545 (18)	0.77853 (16)	0.61765 (13)	0.0728 (5)	

### Atomic displacement parameters (Å^2^)

**Table:**

	*U*^11^	*U*^22^	*U*^33^	*U*^12^	*U*^13^	*U*^23^
C1	0.0499 (11)	0.0524 (11)	0.0330 (9)	−0.0199 (9)	−0.0005 (8)	−0.0002 (8)
C2	0.0399 (9)	0.0389 (9)	0.0298 (8)	−0.0142 (7)	−0.0017 (7)	−0.0034 (7)
C3	0.0377 (9)	0.0347 (8)	0.0327 (8)	−0.0095 (7)	−0.0074 (7)	−0.0035 (6)
C4	0.0312 (8)	0.0334 (8)	0.0311 (8)	−0.0097 (6)	−0.0052 (6)	−0.0039 (6)
C5	0.0305 (8)	0.0460 (10)	0.0352 (9)	−0.0059 (7)	−0.0080 (7)	−0.0036 (7)
C6	0.0310 (8)	0.0433 (9)	0.0284 (8)	−0.0123 (7)	−0.0040 (6)	−0.0072 (7)
C7	0.0283 (8)	0.0337 (8)	0.0280 (7)	−0.0097 (6)	−0.0042 (6)	−0.0068 (6)
C8	0.0308 (8)	0.0441 (10)	0.0324 (8)	−0.0124 (7)	0.0007 (6)	−0.0076 (7)
C9	0.0274 (8)	0.0474 (10)	0.0416 (9)	−0.0031 (7)	−0.0069 (7)	−0.0030 (7)
C10	0.0566 (13)	0.0637 (13)	0.0801 (15)	−0.0067 (10)	−0.0388 (12)	−0.0093 (11)
C11	0.0415 (10)	0.0579 (11)	0.0347 (9)	−0.0089 (8)	−0.0026 (7)	0.0040 (8)
C12	0.0452 (10)	0.0649 (12)	0.0352 (9)	−0.0233 (9)	−0.0109 (8)	0.0091 (8)
C13	0.0413 (10)	0.0584 (12)	0.0375 (10)	−0.0115 (9)	−0.0004 (8)	−0.0009 (8)
C14	0.0329 (9)	0.0750 (13)	0.0337 (9)	−0.0191 (9)	0.0004 (7)	−0.0023 (8)
C15	0.0365 (9)	0.0342 (8)	0.0304 (8)	−0.0091 (7)	−0.0076 (6)	−0.0026 (6)
C16	0.0298 (8)	0.0377 (9)	0.0365 (9)	−0.0083 (7)	−0.0045 (7)	−0.0047 (7)
C17	0.0368 (9)	0.0324 (8)	0.0288 (8)	−0.0115 (7)	−0.0022 (6)	−0.0045 (6)
C18	0.0458 (10)	0.0395 (9)	0.0358 (9)	−0.0133 (8)	0.0042 (7)	−0.0034 (7)
C19	0.0483 (11)	0.0515 (11)	0.0385 (10)	−0.0105 (9)	−0.0161 (8)	0.0025 (8)
N1	0.0549 (9)	0.0520 (9)	0.0302 (7)	−0.0205 (7)	−0.0041 (6)	−0.0018 (6)
N2	0.0332 (8)	0.0654 (10)	0.0276 (7)	−0.0139 (7)	−0.0012 (6)	0.0003 (7)
N3	0.0296 (7)	0.0550 (9)	0.0329 (7)	−0.0097 (6)	−0.0051 (6)	−0.0026 (6)
N4	0.0301 (7)	0.0423 (8)	0.0284 (7)	−0.0104 (6)	−0.0035 (5)	−0.0029 (6)
N5	0.0282 (7)	0.0410 (8)	0.0296 (7)	−0.0082 (6)	−0.0036 (5)	−0.0040 (5)
O1	0.0535 (9)	0.0927 (11)	0.0404 (7)	−0.0267 (8)	0.0077 (6)	0.0014 (7)
OW1	0.0568 (9)	0.0730 (10)	0.0537 (9)	−0.0144 (8)	−0.0185 (7)	0.0068 (7)
O2	0.0631 (10)	0.0786 (10)	0.0395 (7)	−0.0200 (8)	−0.0073 (7)	0.0145 (7)
OW2	0.0719 (11)	0.0748 (11)	0.0624 (10)	−0.0340 (9)	0.0177 (8)	−0.0015 (8)
O3	0.0469 (8)	0.0571 (8)	0.0416 (7)	−0.0086 (6)	−0.0123 (6)	0.0107 (6)
OW3	0.0943 (14)	0.0840 (13)	0.0773 (12)	−0.0392 (11)	−0.0119 (11)	0.0024 (10)
OW4	0.0926 (13)	0.0772 (11)	0.0517 (9)	−0.0234 (10)	0.0125 (9)	−0.0031 (9)
O4	0.0650 (10)	0.0936 (12)	0.0312 (7)	−0.0228 (8)	−0.0001 (6)	0.0087 (7)
O5	0.0412 (8)	0.0692 (9)	0.0569 (8)	−0.0126 (7)	0.0083 (6)	0.0090 (7)
O6	0.0667 (10)	0.0878 (11)	0.0363 (7)	−0.0113 (9)	−0.0163 (7)	0.0145 (7)
O7	0.0492 (9)	0.0929 (12)	0.0579 (9)	0.0087 (8)	−0.0165 (7)	0.0124 (8)

### Geometric parameters (Å, °)

**Table:**

C1—O1	1.219 (2)	C12—H12B	0.9700
C1—O2	1.320 (2)	C13—N1	1.489 (2)
C1—C2	1.475 (2)	C13—C14	1.503 (3)
C2—C8	1.368 (2)	C13—H13A	0.9700
C2—C3	1.430 (2)	C13—H13B	0.9700
C3—O3	1.268 (2)	C14—N2	1.462 (2)
C3—C4	1.439 (2)	C14—H14A	0.9700
C4—C7	1.400 (2)	C14—H14B	0.9700
C4—C5	1.401 (2)	C15—C16	1.393 (2)
C5—N3	1.311 (2)	C15—C17^i^	1.406 (2)
C5—H5A	0.9300	C15—C19	1.525 (2)
C6—N4	1.339 (2)	C16—C17	1.392 (2)
C6—N2	1.350 (2)	C16—H16	0.917 (19)
C6—N3	1.367 (2)	C17—C15^i^	1.406 (2)
C7—N4	1.3307 (19)	C17—C18	1.525 (2)
C7—N5	1.3834 (19)	C18—O5	1.230 (2)
C8—N5	1.345 (2)	C18—O4	1.275 (2)
C8—H8A	0.9300	C19—O7	1.213 (2)
C9—N5	1.487 (2)	C19—O6	1.285 (2)
C9—C10	1.499 (3)	N1—H1A	0.9000
C9—H9A	0.9700	N1—H1B	0.9000
C9—H9B	0.9700	OW1—HW1A	0.878 (15)
C10—H10A	0.9600	OW1—HW1B	0.854 (15)
C10—H10B	0.9600	O2—H2A	0.986 (16)
C10—H10C	0.9600	OW2—HW2A	0.842 (16)
C11—N2	1.451 (2)	OW2—HW2B	0.860 (16)
C11—C12	1.507 (3)	OW3—HW3A	0.849 (16)
C11—H11A	0.9700	OW3—HW3B	0.850 (17)
C11—H11B	0.9700	OW4—HW4A	0.871 (16)
C12—N1	1.491 (2)	OW4—HW4B	0.812 (16)
C12—H12A	0.9700	O6—H6A	0.924 (17)
			
O1—C1—O2	120.65 (17)	N1—C13—C14	110.52 (15)
O1—C1—C2	123.66 (18)	N1—C13—H13A	109.5
O2—C1—C2	115.69 (16)	C14—C13—H13A	109.5
C8—C2—C3	120.28 (14)	N1—C13—H13B	109.5
C8—C2—C1	119.08 (16)	C14—C13—H13B	109.5
C3—C2—C1	120.64 (15)	H13A—C13—H13B	108.1
O3—C3—C2	122.75 (15)	N2—C14—C13	109.98 (14)
O3—C3—C4	121.96 (15)	N2—C14—H14A	109.7
C2—C3—C4	115.29 (14)	C13—C14—H14A	109.7
C7—C4—C5	115.19 (14)	N2—C14—H14B	109.7
C7—C4—C3	121.64 (14)	C13—C14—H14B	109.7
C5—C4—C3	123.17 (15)	H14A—C14—H14B	108.2
N3—C5—C4	123.88 (15)	C16—C15—C17^i^	117.61 (15)
N3—C5—H5A	118.1	C16—C15—C19	113.70 (15)
C4—C5—H5A	118.1	C17^i^—C15—C19	128.68 (15)
N4—C6—N2	117.33 (15)	C17—C16—C15	124.81 (15)
N4—C6—N3	126.49 (14)	C17—C16—H16	120.7 (11)
N2—C6—N3	116.17 (14)	C15—C16—H16	114.4 (11)
N4—C7—N5	117.27 (13)	C16—C17—C15^i^	117.58 (14)
N4—C7—C4	123.16 (14)	C16—C17—C18	113.93 (15)
N5—C7—C4	119.57 (14)	C15^i^—C17—C18	128.49 (15)
N5—C8—C2	123.73 (15)	O5—C18—O4	123.21 (16)
N5—C8—H8A	118.1	O5—C18—C17	118.33 (16)
C2—C8—H8A	118.1	O4—C18—C17	118.46 (16)
N5—C9—C10	111.63 (15)	O7—C19—O6	121.26 (18)
N5—C9—H9A	109.3	O7—C19—C15	119.71 (17)
C10—C9—H9A	109.3	O6—C19—C15	119.03 (17)
N5—C9—H9B	109.3	C13—N1—C12	111.63 (13)
C10—C9—H9B	109.3	C13—N1—H1A	109.3
H9A—C9—H9B	108.0	C12—N1—H1A	109.3
C9—C10—H10A	109.5	C13—N1—H1B	109.3
C9—C10—H10B	109.5	C12—N1—H1B	109.3
H10A—C10—H10B	109.5	H1A—N1—H1B	108.0
C9—C10—H10C	109.5	C6—N2—C11	123.41 (14)
H10A—C10—H10C	109.5	C6—N2—C14	122.94 (15)
H10B—C10—H10C	109.5	C11—N2—C14	112.99 (13)
N2—C11—C12	110.08 (15)	C5—N3—C6	115.45 (14)
N2—C11—H11A	109.6	C7—N4—C6	115.83 (14)
C12—C11—H11A	109.6	C8—N5—C7	119.48 (14)
N2—C11—H11B	109.6	C8—N5—C9	120.08 (13)
C12—C11—H11B	109.6	C7—N5—C9	120.43 (13)
H11A—C11—H11B	108.2	HW1A—OW1—HW1B	101.7 (17)
N1—C12—C11	110.68 (14)	C1—O2—H2A	105.2 (15)
N1—C12—H12A	109.5	HW2A—OW2—HW2B	107 (2)
C11—C12—H12A	109.5	HW3A—OW3—HW3B	106 (2)
N1—C12—H12B	109.5	HW4A—OW4—HW4B	109 (2)
C11—C12—H12B	109.5	C19—O6—H6A	111.5 (16)
H12A—C12—H12B	108.1		

Symmetry codes: (i) −*x*, −*y*+2, −*z*+1.

### Hydrogen-bond geometry (Å, °)

**Table:**

*D*—H···*A*	*D*—H	H···*A*	*D*···*A*	*D*—H···*A*
O6—H6A···O4^i^	0.92 (2)	1.47 (2)	2.392 (2)	178 (3)
N1—H1A···OW1^ii^	0.90	2.08	2.952 (2)	164
N1—H1A···O6^ii^	0.90	2.56	3.022 (2)	113
N1—H1B···OW2^ii^	0.90	1.82	2.717 (2)	176
OW1—HW1A···O1^iii^	0.88 (2)	1.98 (2)	2.780 (2)	150 (2)
OW1—HW1B···O3	0.85 (2)	2.38 (2)	3.041 (2)	135 (2)
OW1—HW1B···O7	0.85 (2)	2.57 (2)	3.204 (2)	132 (2)
OW1—HW1B···O6	0.85 (2)	2.59 (2)	3.084 (2)	118 (2)
O2—H2A···O3	0.99 (2)	1.56 (2)	2.5013 (19)	159 (2)
OW2—HW2B···OW4^iv^	0.86 (2)	1.86 (2)	2.703 (3)	168 (3)
OW2—HW2A···O5^iv^	0.84 (2)	1.98 (2)	2.819 (2)	173 (3)
OW3—HW3A···OW1^v^	0.85 (2)	1.93 (2)	2.770 (2)	169 (3)
OW3—HW3B···O2^vi^	0.85 (2)	2.17 (2)	3.011 (3)	172 (3)
OW4—HW4A···O4^vii^	0.87 (2)	1.94 (2)	2.782 (2)	163 (3)
OW4—HW4B···OW3	0.81 (2)	1.97 (2)	2.775 (3)	174 (3)

Symmetry codes: (i) −*x*, −*y*+2, −*z*+1; (ii) *x*, *y*, *z*−1; (iii) *x*+1, *y*, *z*; (iv) −*x*+1, −*y*+2, −*z*+1; (v) −*x*+1, −*y*+1, −*z*+1; (vi) *x*+1, *y*, *z*−1; (vii) −*x*+1, −*y*+2, −*z*.
